# Epidemiology and burden of illness of seasonal influenza among the elderly in Japan: A systematic literature review and vaccine effectiveness meta‐analysis

**DOI:** 10.1111/irv.12814

**Published:** 2020-09-30

**Authors:** Kiyosu Taniguchi, Shunya Ikeda, Yuriko Hagiwara, Daisuke Tsuzuki, Marwa Klai, Yoko Sakai, Bruce Crawford, Joshua Nealon

**Affiliations:** ^1^ Department of Clinical Research National Mie Hospital Tsu Japan; ^2^ Department of Public Health School of Medicine International University of Health and Welfare Narita Japan; ^3^ Sanofi Pasteur Tokyo Japan; ^4^ Department of Health Economics and Outcomes Research Graduate School of Pharmaceutical Sciences The University of Tokyo Tokyo Japan; ^5^ Sanofi Pasteur Lyon France; ^6^ Syneos Health Tokyo Japan

**Keywords:** economic burden, elderly, epidemiology, Japan, meta‐analysis, seasonal influenza, systematic literature review, vaccine effectiveness

## Abstract

**Background:**

Elderly populations are particularly vulnerable to influenza and often require extensive clinical support. In Japan, nationwide passive surveillance monitors seasonal influenza but does not capture the full disease burden. We synthesized existing evidence on the epidemiology, vaccine effectiveness (VE), and economic burden of seasonal influenza in the elderly population.

**Methods:**

PubMed, EMBASE, and ICHUSHI were searched for articles on seasonal influenza in Japan, published between 1997 and 2018, in English or Japanese. Grey literature was also assessed. A random‐effects meta‐analysis characterized VE of influenza vaccines among studies reporting this information.

**Results:**

Of 1,147 identified articles, 143 met inclusion criteria. Reported incidence rates varied considerably depending on study design, season, study setting and, most importantly, case definition. In nursing homes, the maximum reported attack rate was 55.2% and in the 16 articles reporting mortality rates, case fatality rates varied from 0.009% to 14.3%. Most hospitalizations were in people aged >60; healthcare costs were partially mitigated by vaccine administration. Meta‐analysis estimated overall VE of 19.1% (95% CI: 2.3% ‐ 33.0%) with a high proportion of heterogeneity (I^2^: 89.1%). There was a trend of lower VE in older people (40.1% [−57.3‐77.2] in the <65 group; 12.9% [−8.0‐29.8] in those 65; *P* = .21).

**Conclusions:**

Despite differences between studies that make comparisons challenging, the influenza burden in elderly Japanese is significant. While vaccines are effective, current vaccination programs offer suboptimal protection. Health economic data and cost‐effectiveness analyses were limited and represent areas for policy‐relevant future research.

## 1 INTRODUCTION

Seasonal influenza is an acute respiratory illness caused by influenza type A and B viruses which are clustered into seasonal outbreaks typically lasting 8‐10 weeks from late autumn to early spring.^1^ These annual epidemics are responsible for approximately 3 to 5 million cases of severe illness and 290 000 to 650 000 respiratory deaths worldwide,^2^ and thereby place unwelcome pressure on healthcare systems. Morbidity and mortality are disproportionately high among the elderly, the very young, and people with certain chronic diseases who are therefore targeted for influenza vaccination.^3^

In common with other temperate countries, Japan suffers seasonal outbreaks and reports the second‐highest number of cases in the World Health Organization (WHO) Western Pacific Region, after China.^4^ According to the real‐time pharmacy surveillance program jointly run by several Japanese medical associations and the National Institute of Infectious Disease (NIID) surveillance, there were an estimated 14.3 million influenza cases in Japan in the 2017/2018 season, representing the highest number since the system's inception in 2008^5^, ^6^; and 12.0 million in the 2018/19 season.^5^ Information on influenza mortality is scarce but in a global modeling estimate, the influenza‐associated respiratory mortality rate in Japan was estimated at 0.2, 3.5, and 27.5 per 100 000 individuals aged <65 years, 65‐74 years, and ≥75 years, respectively.^7^ Considering Japan's aging population – the proportion aged ≥65 is projected to rise from 28.4% in 2019 to 35.3% in 2040 – without more effective prevention, the influenza disease burden is likely to increase.^8^

National influenza surveillance data, maintained by the National Institute of Infectious Disease (NIID) and others, are available in Japan, but as with other countries these systems are designed for epidemic detection, to monitor epidemiological trends and to detect circulating influenza viruses rather than to fully measure disease burden.^9^ Dedicated epidemiological studies are therefore often conducted to improve the understanding of disease incidence, severity, and risk factors, to inform health policy.

Annual vaccination against seasonal influenza using quadrivalent influenza vaccines (QIV), which include both influenza B lineages, is recommended for elderly and other high‐risk population groups including those with chronic diseases, residents receiving home‐ and facility‐based care, their regular contacts and healthcare workers.^10^, ^11^, ^12^ One limitation to this preventative health strategy is that the vaccine effectiveness (VE) of influenza vaccination has been shown to decline in individuals aged over 65‐70 years old, a consequence of age‐associated immune dysfunction (immunosenescence),^13^ which is one reason the WHO has advocated for the development of improved influenza vaccines.^14^ Antiviral therapies are commonly used in Japan both therapeutically and, in some instances, for influenza prophylaxis, particularly in residential care and similar facilities in which influenza outbreak risk is highest.^15^

Assessing the cost‐effectiveness of vaccination programs requires both disease costs and information on vaccine performance at averting them. Current influenza vaccination programs have generally been shown to be cost‐effective in Japan,^16^ but robust economic data may be needed as inputs in future health economic analyses of new vaccines. Globally, influenza vaccine VE is affected by virus type and subtype, antigenic match between vaccine and circulating strains, the age and health status of vaccine recipients, and the time between vaccine receipt and infection.^17^ Due to the heterogeneity of VE measurement and associated challenges in interpreting these data, meta‐analytical approaches have been employed to understand vaccine performance and its variation across populations, seasons, and strains.^18^

We conducted a systematic review of literature published in English or Japanese to synthesize evidence describing seasonal influenza epidemiology, prevention, and health economics in Japan, and to identify important gaps. We then conducted a meta‐analysis to estimate reported VE and describe sources of heterogeneity in vaccine performance.

## 2 METHODS

### 2.1 Search strategy

Articles published in English or Japanese between January 1, 1997 and November 20, 2018 were screened from PubMed, EMBASE, and ICHUSHI literature databases. Search strings were related to seasonal influenza in the Japanese population and included: “influenza”; “epidemiology” [and related terms]; “health economics” [and related terms]; “vaccine*”; “effectiveness”; “Japan”; “adult”;as MeSH or Emtree terms; or in the title and abstract of articles (detailed in Supplementary Tables 1‐3). The references of identified systematic reviews and meta‐analyses were screened for further citations which were subject to the same selection process. Grey literature from local medical associations and government institutions in Japan were also assessed. These sources included: National Institute of Infectious Diseases, Ministry of Health, Labor, and Welfare (MHLW) Grant system, Japan Physicians Association (JPA), Online Receipt Computer Advantage (ORCA) surveillance, and MHLW Vaccine Committee.

**Table 1:** Characteristics of studies examining influenza incidence and vaccine effectiveness in different age groups according to the studied season, target population, outcome measure, and vaccine effectiveness

Authors	Influenza season	Population	*Influenza definition* * and incidence	Reported vaccine effectiveness (VE)
Deguchi and Nishimura*, † ^,^ ^57^	1998/1999	Nursing home residents; n = 22,462; mean age vacc vs unvacc = 82.6 vs 81.4	*LCI* in vacc: 6/10,739 (2.4%), unvacc: 694/11,723 (5.9%)	Incidence in vacc significantly lower (*P* < .05)
Imaizumi and Sakai*, † ^,^ ^63^	1998/1999	Nursing home residents; n = 74; mean age = 82.3 28% vacc	*RTI* reported in 41/74 (55.4%) Vacc (52.8%), unvacc (50.9%)	NR
Kobashi et al* ^,^ ^93^	1998‐2000	Community‐acquired pneumonia; n = 84; mean age = 78	*LCI* in 8/84 (9.5%)	NR
Saito et al* ^,^ ^25^	1998/1999	Nursing home residents; n = 699; mean age vacc vs unvacc = 81.3 vs 83.5 45.4% vacc	*ILI* in 170/699 (24.3%)	RR: 0.58 (*P* < .01)
	1999/2000	Nursing home residents; n = 930; mean age vacc vs unvacc = 82.7 vs 83.4 79.9% vacc	*ILI* in 82/930 (8.8%)	RR: 1.22 (*P* = .6)
Ikematsu et al* ^,^ ^75^	1999	Nursing home residents; n = 264; mean age = 82.2	*ILI* in 112/264 (42.4%)	NR
Fujimoto et al^94^	1999	Nursing home residents and workers; age ≥20 = 333; ≥40 = 288 1.6% vacc	*RDT* positive in 12.5% of residents, 35.1% of workers	NR
Suzuki et al*, † ^,^ ^24^	1999	Nursing home residents; n = 440; mean age vacc vs unvacc = 80.3 vs 80.5	*ILI* in vacc: 39/226 (17.3%), unvacc: 60/214 (28.0%) *Pneumonia* in vacc: 2/226 (0.9%), unvacc: 13/214 (6.0%) *LCI* in 28/78 (35.9%)	NR
Takahashi*, † ^,^ ^26^	1999	Nursing home residents; n = 96; mean age = NR 22% vacc	*ILI* in 53/96 (55.2%)	NR
Nishi et al^95^	1999/2000	Healthcare workers; n = 727; age ≥20 18.2% vacc	*ILI* in vacc: 9.9% Unvacc: 19.7%	NR
Kawai et al ^,^ ^96^	2000	Patients from internet surveillance by different age groups; n = 5,201; age ≥20	*CDI* in [age 20s] 1,356, [30s] 1,317, [40s] 801, [50s] 837, [60s] 551, [70s] 260, [≥80] 79	NR
Kobashigawa et al*, † ^,^ ^39^	2000/2001	Patients with rheumatoid arthritis; n = 3,661; mean age = 59 12.2% vacc	*SRIof ILI* in 339/3,661 (9.6%)	VE: 28.8% (95% CI: NR)
	2001/2002	Patients with rheumatoid arthritis; n = 4,628; mean age = 59 17.0% vacc	*SRI* in 351/4,628 (7.8%)	VE: 17.9% (95% CI: NR)
	2002/2003	Patients with rheumatoid arthritis; n = 4,939; mean age = 59 20.9% vacc	*SRI* in 319/4,949 (6.6%)	VE: 16.3% (95% CI: NR)
	2006/2007	Patients with rheumatoid arthritis n = 5,175; mean age = 60 38.7% vacc	*SRI* in 164/5,175 (3.4%)	VE: 10.4% (95% CI: NR)
Shijubo et al* ^,^ ^29^	2001	Nursing home residents; n = 68; mean age = 81	*RDT* positive in 28/68 (41.2%)	NR
Kawai et al*, † ^,^ ^97^	2001/2002	Elderly population from internet surveillance; n = 4,423; age ≥65	*RDT* positive in vacc once: 5/3,140, vacc twice: 0/380, unvacc = 2/903 *ILI* in vacc once 16/3140, vacc twice: 3/380, unvacc: 6/903	VE for influenza: vaccinated once vs twice = 28.1 (95% CI: 3.7, 71.0) vs 100 (95% CI: NR) VE for ILI: vaccinated once vs twice = 23.3 (95% CI: 0.1, 47.2) vs −18.8 (95% CI: NR)
Wakita et al* ^,^ ^98^	2001/2002	Nursing home residents; n = 116; mean age = NR 58.6% vacc	*LCI* in 28/116 (24.1%)	NR
	2002/2003	Nursing home residents; n = 127; mean age = NR 79.5% vacc	*ILI* in 0/127	NR
Hara et al*, † ^,^ ^56^	2002/2003	Nursing home residents, n = 114; age ≥66	*RDT* positive in 8/114 (7.0%)	VE = 36 (95% CI: NR)
Kobayashi et al*, † ^,^ ^27^	2002/2003	Nursing home residents; n = 424; mean age = 83.5 88% vacc	*ILI* in vacc: 12/373, unvacc: 1/51 *Pneumonia* in vacc: 13/373, unvacc: 3/51	NR
Okamoto et al* ^,^ ^99^	2002	Elderly population from claim data; n = 10,530; mean age vacc vs unvacc = 75.3 vs 73.5 30% vacc	*CDI* in vacc: 6/3589, unvacc: 34/6302 *Pneumonia* in vacc: 14/3589, unvacc: 44/6302	NR
Ohbayashi et al* ^,^ ^100^	2002	Community residents by different age groups; n = 77; age ≥20	*RDT* positive in [age: 20s] 17/71 (23.9%); [30s] 15/62 (24.2%); [40s] 10/33 (30.3%); [50‐64] 13/46 (28.3%); [65‐74] 9/48 (18.8%); [≥75] 13/52 (25%)	NR
	2003	Community residents by different age groups; n = 64; age ≥20	*RDT* positive in [age 20s] 18/67 (26.9%); [30s] 16/51 (31.4%); [40s] 9/25 (36.0%); [50‐64] 6/27 (22.2%); [65‐74] 10/25 (40.0%); [≥75] 5/39 (12.8%)	NR
	2004	Community residents by different age groups; n = 187; age ≥20	*RDT* positive in [age 20s] 37/132 (28.0%); [30s] 40/126 (31.7%); [40s] 30/83 (36.1%); [50‐64] 33/95 (34.7%); [65‐74] 20/58 (34.5%); [≥75] 24/87 (27.6%)	NR
Yamanaka et al^101^	2002	HIV‐1 infected patients; n = 328; mean age vacc vs unvacc = 41 vs 40 79.9% vacc	*ILI + RDT/LCI* positive in vacc: 16/262 (6.1%), unvacc: 14/66 (21.2%)	NR
Ide et al*, † ^,^ ^48^	2002/2003	Nursing home residents; n = 89; mean age = 84.5 84% vacc	*ILI* in vacc: 12/75 (12.0%), unvacc: 5/14 (35.7%)	HR = 0.41 (*P* = .095)
	2003/2004	Nursing home residents; n = 92; mean age = 72.4 13% vacc	*ILI* in vacc: 1/12 (8.3%), unvacc: 8/80 (10.0%)	HR = 0.59 (*P* = .619)
Ito et al^102^	2002/2003	Healthcare workers; n = 366; mean age vacc vs unvacc = 35.5 vs 35.7 64.8% vacc	*RDT* positive in vacc: 8/237 (3.4%), unvacc: 11/129 (8.5%)	NR
Kawai et al*, † ^,^ ^103^	2002/2003	Community residents by different age groups; n = NR; age ≥20 In those aged ≥65, 81.7% vacc once, 4.3% vacc twice	*ILI (including RDT positive)* in [age 20s] vacc: 4.4%, unvacc: 13.2% [30s] vacc: 2.4%, unvacc: 9.8% [40s] vacc: 2.1%, unvacc: 8.6% [50s] vacc: 1.4%, unvacc: 2.5% [60s] vacc: 1.2%, unvacc: 1.6% [70s] vacc 1.1%, unvacc: 1.6% [≥80] vacc 1.5%, unvacc: 0%	VE for influenza [≥65] once vs twice = 14.4 (95% CI: 4.5, 24.3) vs −23.0 (95% CI: NR) VE for ILI [≥65] once vs twice = −2.7 (95% CI: NR) vs −9.3 (95% CI: NR)
Kuchibiro et al* ^,^ ^104^	2002/2003	Community residents by different age groups; n = 775; age ≥21	*RDT* positive in [age 20s] 52/132 (39.4%); [30s] 55/141 (39.0%); [40s] 38/99 (38.4%); [50s] 32/94 (34.0%); [60s] 24/77 (31.2%); [70s] 40/128 (31.3%); [≥81] 29/104 (27.9%)	NR
Moriguchi et al* ^,^ ^105^	2002/2003	Elderly community residents; n = 61; age ≥65 100% vacc	*CDI* in 1.8%	NR
Washio et al* ^,^ ^106^	2002/2003	Nursing homes; n = 409; age ≥80	*ILI*: 28.1% of institutions reported ILI incidence among users	NR
Hashimoto et al* ^,^ ^107^	2002	Estimated number of influenza patients in Japan; aged ≥20	*CDI incidence* [age 20s] 820,000; [30s] 840,000; [40s] 440,000; [50s] 270,000; [60s] 160,000; [≥70] 130,000	NR
	2003	Estimated number of influenza patients in Japan; age ≥20	*CDI incidence*: [age 20s] 1,300,000, [30s] 1,210,000, [40s] 760,000, [50s] 550,000; [60s] 350,000, [≥70] 340,000	NR
	2004	Estimated number of influenza patients in Japan; age ≥20	*CDI incidence*: [age 20s] 1,100,000; [30s] 980,000; [40s] 610,000; [50s] 390,000; [60s] 270,000; [≥70] 300,000	NR
Washio et al*, † ^,^ ^44^	2002‐2005	Nursing home residents; n = 1257; mean age = NR 91% vacc	*ILI* in vacc: 108/1,150 (9.4%), unvacc: 8/107 (7.5%) *Pneumonia* in vacc: 32/1,150 (2.8%), unvacc: 3/107 (2.8%)	HR of ILI = 1.20 (95% CI: 0.58, 2.46] HR of pneumonia = 0.93 (95% CI: 0.28, 3.02)
Chiya et al^*†,^ ^108^	2003	Nursing home residents; n = 80; mean age = NR 86% vacc	*RDT* in vacc: 35%, unvacc: 36%	NR
Nishi et al* ^,^ ^95^	2003	Healthcare workers; n = 684; age ≥20 43% vacc	*SRI*: [20s] 4.7%, [30s] 9.2%, [40s] 4.8%, [50s] 6.5%, [60s] 0%	NR
Fujita et al^109^	2003/2004	Healthcare workers; n = 830; mean age vacc vs unvacc = 32.1 vs 35.1 62% vacc	*SRI* in vacc: 1.4%, unvacc: 1.9%	Influenza incidence was not significantly different
	2004/2005	Healthcare workers; n = 850; mean age vacc vs unvacc = 32.6 vs 34.5 82.7% vacc	*SRI* in vacc: 5.9%, unvacc: 10.9%	Influenza incidence was significantly different
Hara et al*, †, ^59^	2003/2004	Elderly community residents; n = 4,787; age ≥65 86% vacc	*CDI* in vacc: 18/3,169 (0.6%), unvacc: 10/1,540 (0.6%) *ILI* in vacc: 20/3,169 (0.6%), unvacc: 22/1,540 (1.4%)	NR
Kawai et al*, † ^,^ ^71^	2003/2004	Community residents by different age groups; n = NR; age ≥20	*RDT* positive in [age 20s] vacc: 1.7%, unvacc: 3.1% [30s] vacc: 1.3%, unvacc: 1.9%, [40s] vacc: 1.1%, unvacc: 3.1%, [50s] vacc: 1.4%, unvacc: 1.5%, [60s] vacc: 0.6%, unvacc: 0.4%, [70s] vacc: 0.6%, unvacc: 0.2%; [80s] vacc: 0.7%, unvacc: 0.4% *ILI*: [20s] vacc: 2.3%, unvacc: 4.0%, [30s] vacc: 1.4%, unvacc: 2.2%; [40s] vacc: 1.2%, unvacc: 3.5%, [50s] vacc: 1.6%, unvacc: 2.2%; [60s] vacc: 1.0%, unvacc: 0.6%; [70s] vacc: 0.9%, unvacc: 0.4%; [80s] vacc: 1.1%, unvacc: 1.2%	VE for influenza [≥65] <0 (95% CI: NR)
Ozasa et al* ^,^ ^110^	2003/2004	Non‐institutionalized elderly; n = 2,301; age ≥65 66.6% vacc	*CDI* in 1.8%	OR for influenza = 0.81 (95% CI: 0.41, 1.57)
Kanaoka et al* ^,^ ^111^	2003/2004	Nursing home residents; n = 183; mean age = NR 45.4% vacc	*ILI, CDI, RDT* positive in 22/183 (12.0%)	Correlation between vaccination and influenza outbreak = −0.9 (*P* = .014)
	2004/2005	Nursing home residents; n = 185; mean age = NR 49.7% vacc	*ILI, CDI, RDT* positive in 10/185 (5.4%)	NR
	2005/2006	Nursing home residents; n = 181; mean age = NR 57.5% vacc	*ILI, CDI, RDT* positive in 10/181 (5.5%)	NR
	2006/2007	Nursing home residents; n = 184; mean age = NR 65.8% vacc	*ILI, CDI, RDT* positive in 0/184 (0%)	NR
	2007/2008	Nursing home residents; n = 180; mean age = NR 65.6% vacc	*ILI, CDI, RDT* positive in 2/180 (1.1%)	NR
	2008/2009	Nursing home residents; n = 182; mean age = NR 72.0% vacc	*ILI, CDI, RDT* positive in 2/182 (1.1%)	NR
Usami et al*, ^112^	2004	Elderly community residents received vacc advocacy; n = 1,863; age ≥65 61.3% of intervention group, 53.3% of control group vacc	*Patients with influenza drug prescriptions* in 2/881 (0.2%) with intervention, 11/895 (1.2%) without intervention	NR
Yamauchi et al*, † ^,^ ^113^	2004	Nursing home residents; n = 104; mean age = NR 92.3% vacc	*RDT* positive in vacc: 54/96 (56.3%), unvacc: 2/9 (22.2%)	NR
Maruyama et al* ^,^ ^114^	2004/2005	Community and nursing home residents; n = 108; mean age = 85.0	*LCI* in 12/85 (11.1%)	NR
Kawai et al*, † ^,^ ^72^	2004/2005	Elderly community residents; n = 6,066; age ≥65	*RDT* type A positive in vacc: 29/5,326 (0.5%), unvacc: 6/740 (0.8%) *RDT* type B positive in vacc: 69/5,326 (1.3%), unvacc: 11/740 (1.5%)	VE for type A = 32.8% (95% CI: NR) VE for type B = 12.8% (95% CI: NR)
Yamashita et al^115^	2004/2005	Residents and healthcare workers in nursing home; n = 83; mean age = NR	*CDI, RDT* positive in 11/83 (13.3%)	NR
	2005/2006	Residents and healthcare workers in nursing home; n = 57; mean age = NR	*CDI, RDT* positive in 2/57 (2%)	NR
	2006/2007	Residents and healthcare workers in nursing home; n = 42; mean age = NR	*CDI, RDT* positive in 1/42 (2.4%)	NR
Hamabe et al ^,^ ^116^	2005	Healthcare workers who are receiving oseltamivir as prophylaxis in nursing home; n = 234; age ≥19 83.7% vacc	*RDT* positive in 0/234	NR
Watanabe et al* ^,^ ^117^	2005	Nursing home residents; n = 80; mean age = 80	*RDT* positive in 19/80 (23.8%)	NR
Yamada et al* ^,^ ^118^	2005	Nursing home residents who gargled with tea catechin; n = 124; mean age = 83	*RDT* positive in 6/124 (4.8%)	NR
Kawai et al*, † ^,^ ^73^	2005/2006	Community residents by different age groups; n = NR; age ≥20	*RDT* positive in [20s] vacc: 1.4%, unvacc: 5.3%, [30s] vacc: 1.5%, unvacc: 1.7%, [40s] vacc: 1.3%, unvacc: 3.2%, [50s] vacc: 0.8%, unvacc: 1.6%, [60s] vacc: 0.6%, unvacc: 0.9%, [70s] vacc: 0.3%, unvacc: 1.3%; [≥80] vacc: 0.4%, unvacc: 1.0%	VE [age > 65] = 57.6% (95% CI: NR)
Kudo et al* ^,^ ^119^	2005/2006	Community residents by different age groups; n = 577; age ≥26 29.7% of non‐elderly vacc 73.9% of elderly vacc	*RDT* positive in [age < 65] vacc: 3.3%, unvacc: 5.6%; [≥65] vacc: 0.7%, unvacc: 3.1%	NR
Momiyama et al ^,^ ^120^	2005/2006	Airport workers; n: 1,174; age ≥20	*RDT* positive in 36/1,174	NR
Yamada et al^121^	2005/2006	RCT of gargling with tea catechin extracts; n = 404; mean age catechin group vs control group = 39.6 vs 40.2	*RDT* positive in catechin group: 1%, control group: 2%	NR
Eto et al* ^,^ ^122^	2006/2007	Community residents by different age groups; n = 191; age ≥19 26.6% of participants aged 19‐64 vacc 61.1% of participants aged ≥65 vacc	*RDT* positive in [age 19‐64] vacc: 13/46 (28.3%), unvacc: 71/127 (55.9%) [≥65] vacc: 4/11 (36.4%), unvacc: 4/7 (57.1%)	NR
Kikuchi et al^†^ ^,^ ^34^	2006/2007	Dialysis patients; n: 339; mean age = 62.4 67.3% vacc	*RDT* positive in vacc: 6/228 (2.6%), unacc: 5/112 (4.5%)	NR
Takano et al^*†,^ ^123^	2006	Nursing home residents; n = 58; mean age = 83.9 58.6% vacc	*RDT* positive in 24%	VE: −27.4% (95% CI: NR)
Yoshino et al^33^	2007	Dialysis patients; n = 187; mean age = 63.6 63% vacc	*Undefined influenza*: 4.8%	NR
	2008	Dialysis patients; n = 189; mean age = 64.2 78% vacc	*Undefined influenza*: 2.3%	NR
Washio et al* ^,^ ^124^	2007	Nursing homes; n = 537 institutions; mean age = NR	*ILI*: 28.0% of institutions reported influenza incidence among users	NR
Fujiwara et al* ^,^ ^125^	2007/2008	Community residents by different age groups; n = 3,389; age ≥25	*RDT* positive in [ages 25‐34] 14/316 (4.4%), [35‐44] 21/312 (6.7%), [45‐54] 9/484 (1.9%), [55‐64] 8/633 (0.1%), [65‐74] 12/653 (0.2%), [75‐84] 3/696 (0.4%), [≥85] 3/290 (0.1%)	RR: [25‐34] = 3.23 (*P* = .046); [35‐44] = 2.19 (*P* = .116); [45‐54] = 1.59 (*P* = .728); [55‐64] = 2.54 (*P* = .334); [65‐74] = 3.48 (*P* = .151); [75‐84] = 1.15 (*P* = 1.000); [≥85] = 0.33 (*P* = .722)
Hirose et al* ^,^ ^126^	2007‐2008	Elderly nursing homes; n = 469; mean age = NR	*ILI* in 68/469 (14.5%) of institutions reported influenza incidence among users	No correlation between vaccination and incidence of influenza
Kawai et al*, † ^,^ ^74^	2007/2008	People in different age groups; n = 151; age ≥20	*RDT* positive in [age 20s] vacc: 1.4%, unvacc: 3.2%, [30s] vacc: 1.5%, unvacc: 2%, [40s] vacc: 0.7%, unvacc: 1.1%, [50s] vacc: 0.2%, unvacc: 0.4%, [60s] vacc: 0.6%, unvacc: 0.5%, [70s] vacc: 0.2%, unvacc: 2.1%, [80s] vacc: 0.1%, unvacc: 0%	VE [>65] = 82.8% (95% CI: NR)
Washio et al^†^, ^36^	2008	Dialysis patients; n = 183; mean age 61.8 in ILI cases; 62.0 in non‐ILI cases	*ILI* in vacc: 12/156 (7.7%), unvacc 5/27 (18.5%)	NR
Hirota et al^†^ ^,^ ^127^	2008/2009	Home care elderly; n = 251; age ≥65	NR	Adjusted OR = 1.09 (95% CI: 0.37, 3.15)
Ikematsu et al*, †, ^46^	2008/2009	Elderly people with acute respiratory infection; n = 401; age ≥50	*LCI* in 70/401 (17.5%)	VE = 32.1% (95% CI: −14.9, 59.9)
Hidaka et al^38^	2009	Patients with rheumatoid arthritis; n = 749; age ≥20 60% vacc	*SRI* in 2.5%	NR
Washio et al^35^	2009	Dialysis patients; n = 197 institutions; mean age = NR	*ILI* in 128/197 (65%) of institutions reported ILI incidence among patients	NR
Matsumoto et al^128^	2009/2010	Healthcare workers; n = 196; mean age = 42.7	*CDI* in 17/196 (8.7%) *LCI* in 6/196 (3.1%)	NR
Ohkusa et al^129^	2010/2011	Influenza patients in different age groups from surveillance in one prefecture; n = NR; age ≥20	*Patients with influenza drug prescriptions*: [age 20s] 690 patients, [30s] 787, [40s] 497	NR
Suzuki et al^†^ ^,^ ^130^	2010/2011	Elderly outpatients; n = 60; age ≥50	NR	VE = 52.60 (95% CI: −306.5, 42.7)
Okuno et al^51^	2010‐2015	Influenza‐associated encephalopathy patients from NIID surveillance; n = 102; age ≥18	*Influenza‐associated encephalopathy*: 0.19 cases per 1 ,000,000 population	NR
Mine et al^131^	2012/2013	Patients in an orthopedic hospital; n = 46; mean age = 66.3	*RDT* positive in 23.9%	NR
Shimoda et al* ^,^ ^132^	2012‐2014	Elderly nursing homes; n = 48; mean age = NR	*SRI*: 41.7% of nursing homes reported influenza incidence of type A, 6.3% did type B	NR
Suzuki et al*, † ^,^ ^61^	2012‐2014	Elderly outpatients; n = 814; age ≥65	*LCI* in 42/814 (5.2%)	VE for influenza‐pneumonia = 56.6 (95% CI: 25.8, 74.6)
Tanaka et al* ^,^ ^133^	2012‐2015	Elderly people with and without prophylaxis; n = 506; mean age with = 72, mean age without = 73 75% vacc	*RDT* positive in 22%	Secondary infection rate with vs without prophylaxis: 0.7% vs 9.5% (*P* < .001)
Seki et al^†^ ^,^ ^134^	2013/2014	Elderly outpatients; n = 107, age ≥65	NR	Adjusted VE for type A = −29% (95% CI: −536, 73.8)
	2014/2015	Elderly outpatients; n = 113, age ≥65	NR	Adjusted VE for type A = −56% (95% CI: −325, 42.4)
Umeki et al^40^	2013/2014	Psychiatric hospital inpatients; n = 884; age ≥20 53% vacc	*RDT* positive in 7.4%	NR
Yamada et al^135^	2013/2014	Pregnant women; n = 1,713, mean age = NR)	*SRI* in 87/1,713 (5.1%)	NR
Watanabe et al* ^,^ ^136^	2014‐2017	Elderly people with prophylaxis; n = 440; mean age = 82.2 95% of participants had prophylaxis	*LCI* in 1%	NR
Ishikane et al* ^,^ ^137^	2014/2015	Nursing home residents; n = 338; mean age = NR 85.4% of < 65, 81.8% (>65) vacc	*RDT* positive in [<65] 34/316 (10.8%), 2/22 (9.1%) in aged ≥65	NR
Kariya et al* ^,^ ^138^	2016/2017	Nursing homes; n = 102; mean age = NR	*SRI*: 10.8% in nursing home, 21.1% in healthcare facility	NR
Iikura et al^139^	NR	Adult hospital inpatients; n = 50; mean age = 58	*LCI* in 8/50 (16%)	NR

Abbreviations: CDI, clinically defined influenza; ILI, influenza‐like illness; LCI, laboratory‐confirmed influenza; NR, not reported; OR, odds ratio; RDT, rapid diagnostic test; RR, relative risk; RTI, respiratory tract infection; SRI, self‐reported influenza; unvacc, unvaccinated; vacc, vaccinated.

Self‐reported influenza was collected in surveys.

^*^ Presented influenza/ILI attack rates or estimated number of incidence exclusively for elderly population aged ≥50.

^†^ Reported VE was used in meta‐analysis. If not explicitly reported, VE was calculated using study data.

### 2.2 Study selection criteria

This review included study designs limited to systematic reviews, prospective or retrospective observational studies (evaluating at least 10 patients), randomized controlled trials and economic studies conducted in Japan. All studies describing populations diagnosed with laboratory‐confirmed influenza (LCI) or symptomatic influenza disease described as influenza‐like‐illness (ILI) or similar, which allowed extraction of data in adult (≥18 years) age groups, were included. Animal studies, in vitro/ex vivo studies, gene expression/protein expression studies, laboratory studies, editorials, non‐systematic reviews, conference minutes, and case studies/case series evaluating fewer than 10 patients were excluded. Articles were excluded if the primary focus was not seasonal influenza. Publications that reported data exclusively on safety, treatment options, pharmacokinetics, pharmacodynamics, and/or patient‐reported outcomes other than utility values were also excluded. Due to the variety of different study types included and the challenges in comparing them, studies were included without assessment of their methodological quality. Two researchers independently screened the titles and abstracts of identified studies and then full texts, to assess eligibility. Information from included articles was extracted into a predefined data extraction template which included study characteristics, target population details, and study outcomes.

### 2.3 Meta‐analysis methodology and statistical analysis

Studies describing the relative risk (RR) of developing influenza in vaccine recipients vs non‐recipients were meta‐analyzed to estimate influenza vaccine effectiveness (VE) reported in the Japanese literature overall and stratified into predefined categories. Data were first organized into age‐stratified RR estimates for each of three age groups: 1) <65 years old, 2) 65 years old and 3) mixed ages. Studies reporting data for multiple years were grouped as single estimates. Age‐specific estimates were then categorized according to their study design (cohort vs case control); setting (nursing home/hospitalized vs community); and single or multiple seasons over which the studies were conducted. The dominant circulating influenza type/subtype was defined for each estimate as “H3”, “H1”, or “B” if >50% of viruses characterized by National Epidemiological Surveillance of Infectious Diseases (NESID) data over the seasons of study corresponded to these viruses; or “mixed” if there was no dominant virus.^15^ As B strains dominated in only one season (2004/05), this category was combined with “mixed” for the meta‐analysis.

Adjusted, reported RR and their confidence intervals (CIs) were used for meta‐analysis if provided. Otherwise, RRs and their CIs were calculated from reported data in the article. Four case control studies reported odds ratios (OR). RRs for these studies were estimated, taking the mean baseline risk from the unvaccinated groups of included cohort studies, an approach which resulted in RRs close to original ORs because the outcome is rare (<5%/year; data not shown).^19^ Studies reporting VE from the 2009 pandemic, reporting non‐respiratory outcomes, and those relying on serological criteria or self‐reporting as study outcomes, were excluded. RR estimates for laboratory‐confirmed influenza were used where available.

A random‐effects meta‐analysis which incorporates between‐ and within‐study variance to account for seasonal and other variations across studies, was performed to combine estimates.^20^ To explore heterogeneity, a meta‐regression approach assessed whether study characteristics (subject age; study setting, design or circulating influenza virus subtypes) were explanatory of the overall relative risk. RR estimates were presented as a forest plot and used to estimate VE using the formula RR = 1‐VE. The risk of publication bias from small studies was assessed using the approach proposed by Egger.^21^ Analyses were conducted in Stata v15.0, using the Admetan package.^22^

## 3 RESULTS

**Figure 1:**
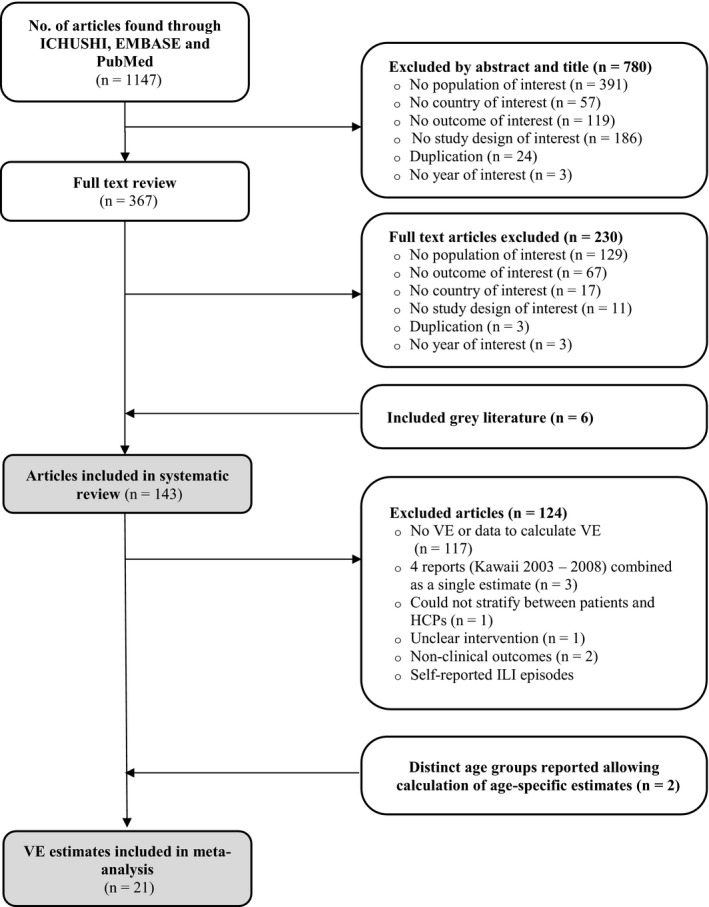
Article Selection Procedure – PRISMA flow chart. PRISMA, Preferred Reporting Items for Systematic Reviews and Meta‐Analyses

A total of 1,147 studies were identified. After reviewing titles and abstracts, 367 full text articles and six grey literature articles were reviewed for relevance, of which 143 met the inclusion criteria. A PRISMA diagram of the selection process is shown in Figure 1.

### 3.1 Epidemiology

#### 3.1.1 Seasonal influenza incidence and hospitalization rate

Of the 74 studies that presented data on seasonal influenza incidence, 36 reported LCI including those using rapid diagnostic kits. The remaining 38 studies reported non‐LCI influenza outcomes such as influenza‐like‐illness (ILI). Most publications originated from studies conducted at single institutions, with a wide geographical distribution across Japan. Forty‐nine studies exclusively surveyed a population aged ≥50 years; the remaining studies included a wider adult population. Twenty‐nine studies included the elderly in institutional settings, either inpatients or nursing home residents, as the main study population with sample sizes ranging from 68 to 22,462 (Table 1).

**Figure 2:**
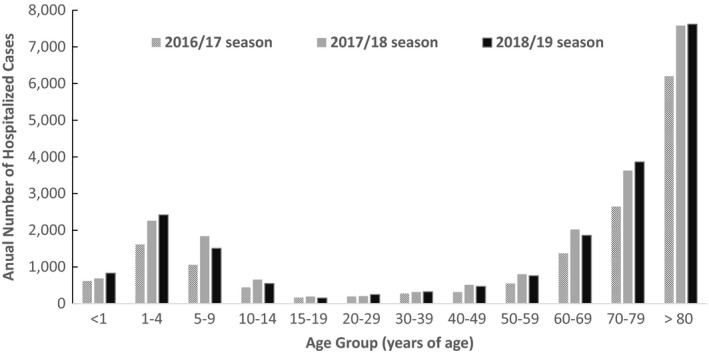
Age distribution of influenza hospitalization in Japan. Data reproduced from National Institute of Infectious Diseases.^23^ CI, confidence interval; ES, effect size; LCI, Laboratory‐confirmed influenza; RTI, respiratory tract infection

Reported incidence rates varied considerably depending on study design, season, study setting and, most importantly, case definitions: non‐specific endpoints such as ILI were much more common than LCI cases. According to national sentinel hospitalization surveillance data from 500 hospitals, the number of hospitalizations with LCI or ILI was highest in the population group aged 70+ (average reported cases from 8800‐11500 per season) followed by those who are aged 0‐4 (2200‐3200 per season); 60‐69 (1300‐2000 per season) over three recent seasons (Figure 2).^23^

Individual studies often reported much higher rates of disease: Suzuki et al reported ILI incidence of 23% in nursing homes during the 1998/1999 season^24^; Saito et al calculated an ILI attack rate of 24.3% among 699 institutionalized patients in the 1998/99 season^25^; and Takahashi et al reported an ILI incidence rate of 55.2% in the 1998/1999 season in nursing homes.^26^ These extremely high attack rates are likely due to extensive social contact in institutional settings, against a backdrop of low vaccine coverage, giving rise to disease outbreaks which are captured by sensitive surveillance and case definitions.^26^

More specific case definitions resulted in lower attack rates. Kobayashi et al required fever of at least 39°C in their study of ILI and/or pneumonia and reported 6.8% of nursing home residents suffered an episode in the 2002/03 season in Hokkaido.^27^

Studies reporting LCI reported lower attack rates than those using syndromic surveillance definitions among the institutionalized elderly. Deguchi et al used a combination of virological and serological diagnosis and a sensitive case definition to estimate an influenza attack rate of 4.2%, and hospitalization rate of 1.3%, in 22,462 nursing home residents in 1998/1999.^28^ Another study conducted in Kumamoto implemented surveillance in a population of nursing homes and convalescent wards of hospital. It used rapid diagnostic kits to confirm influenza and found an overall positivity rate of 24.1% in 2001/02 but 0% in 2002/03 among the institutionalized elderly.^29^

Finally, influenza A viruses were dominant between the seasons of 1998/1999 and 2017/2018.^6^, ^30^, ^31^, ^32^ Of isolated and typed viruses, influenza A comprised >50% of viruses every year from 1997 to 2018, with A/H3 subtypes dominating most often. The proportion of B viruses increased after 2010.

#### 3.1.2 Seasonal influenza incidence in comorbid populations

End‐stage renal disease requiring hemodialysis was the most commonly studied comorbidity associated with influenza, probably because of the recommendation for influenza vaccination in the Guidelines for Standard Hemodialysis Procedure and Prevention of Infection in Maintenance Hemodialysis Facilities (4th edition) in Japan.^33^, ^34^, ^35^, ^36^, ^37^ These patients were recognized to be at high‐risk for infection due to immune dysfunction and receipt of treatment in the same room as other patients. Those who visited a hospital regularly for dialysis had attack rates between 3.2% to 4.8% in 2008 ^33^, ^34^ while another study reported 9.2% ILI incidence during the 2008/2009 season.^36^ Other studies of comorbid patients included investigations of influenza patients with rheumatic diseases,^38^, ^39^ mental illness,^40^ and orthopedic disease ^41^ and while the baseline status of other comorbidities (eg cerebrovascular disease) was occasionally reported, they were not analyzed as subgroups.^42^, ^43^, ^44^

#### 3.1.3 Disease severity

Influenza infections most often resulted in severe and hospitalized outcomes in the elderly Japanese population: from 2011 to 2018, over 60% of influenza hospitalizations occurred in people aged >60.^6^, ^30^, ^45^ Severity and disease progression were also described as a function of setting: a study of 96 nursing home residents with ILI identified that 18.9% developed an asthmatic‐like illness while 9.4% progressed to serious complications such as pneumonia, bronchitis, and heart failure.^26^ A 2011/2012 study in the elderly identified that 50% of influenza hospitalizations developed pneumonia and that older age was an important risk factor for pneumonia (average age with/without pneumonia: 85.3 years vs 71.4 years; *P* < .05).^43^ Assessing severity from a different perspective, a study from the 2008/09 season found influenza led to more severe post‐infection clinical and socio‐economic outcomes than other acute respiratory infections.^46^

#### 3.1.4 Mortality rate and excess mortality

Twenty articles reported mortality rates associated with seasonal influenza,^16^, ^24^, ^61^ 11 of which focused exclusively on the elderly. A large (n = 22 462) cohort study among institutionalized elderly during the 1998/1999 season closely followed and confirmed respiratory episodes, and identified influenza‐related mortality rates of 0.009% and 0.043% in vaccinated and unvaccinated groups of residents, respectively.^28^ Other studies of the institutionalized elderly described mortality rates ranging from 1.3% to 6.9%.^62^, ^63^

Higher mortality rates were observed in complicated influenza cases with additional clinical progression. In a study of adult patients with influenza‐associated pneumonia between 1996 and 2005, 14.3% of those who did not receive neuraminidase inhibitors died, as compared to 4.9% of those who received such treatment.^49^ Most cases and deaths were aged ≥65. Another study identified a mortality rate in influenza‐associated pneumonia from 2003 to 2007 of 9.5% (two deaths out of 21 cases).^64^ High mortality was also associated with influenza‐associated encephalopathy in 2010‐2015; of deaths (n = 14, 13.7%), the MR was highest among patients aged ≥65 years (20%) followed by those aged 50‐64 and 18‐49 (14% and 11%, respectively).^51^

Eight influenza excess mortality studies consistently estimated highest mortality in older people, and the highest death rates in the oldest individuals.^55^, ^65^, ^66^, ^67^, ^68^, ^69^, ^70^ Takahashi et al modeled Ministry of Health vital demographic statistics (which included ICD codes for each cause of death) from 1987 through 2005 and showed that 85%‐90% of the total influenza excess mortality was in the elderly group (aged ≥65 years). Nationwide excess mortality was estimated as 16 000, 23 000, 34 000, 22 000, 44 000 in 1990, 1993, 1995, 1997, and 1999 respectively with increasing trend in seasons where both type A and B virus were circulating.^65^ Nationwide estimates from 1952 to 2009 also showed the highest influenza excess mortality in the elderly (14.81 per 100,000 in those aged ≥65 y ears). Of interest, this study demonstrated that only 7% ~ 18% of total deaths attributable to influenza was recorded as such in health statistics.^66^ In terms of variability in excess mortality, a multi‐year NIID study, calculating excess mortality over many seasons, estimated a peak in influenza deaths at more than 35,000 in the 1998/1999 season.^70^ After 2004/2005, the excess mortality did not exceed 10,000.

### 3.2 Vaccine effectiveness

Twenty‐six studies focused on VE (Table 1) using cohort and case control designs including a series of longitudinal VE studies, conducted between 2003 and 2008, using Japan Physicians Association (JPA) surveillance data.^71^, ^72^, ^73^, ^74^ VE varied according to institution type, age, geographic location, and study period. For example, a survey of 89 elderly residents at a long‐term care health facility demonstrated statistically significant VE against ILI during the 2002/2003 season, an effect which disappeared in 2003/2004,^48^ perhaps due to immunity in unvaccinated individuals conferred after infection with a similar virus in 2002/2003. VE seemed to vary by age and methodology; data collected from 46 medical institutions via online survey showed a significant benefit of vaccination for patients aged 70‐79, but not in those aged 50 ‐ 59, 60 ‐ 69, and ≥80 year‐olds, during the 2005/2006 season.^73^ In contrast, a prospective study involving laboratory‐confirmed influenza in elderly nursing home residents demonstrated a significantly lower influenza attack rate (2.4% vs 5.9%) and mortality rates (0.009% vs 0.043%) in the vaccinated vs unvaccinated groups during the 1998/1999 season.^28^

#### 3.2.1 Meta‐analysis of vaccine effectiveness

**Figure 3:**
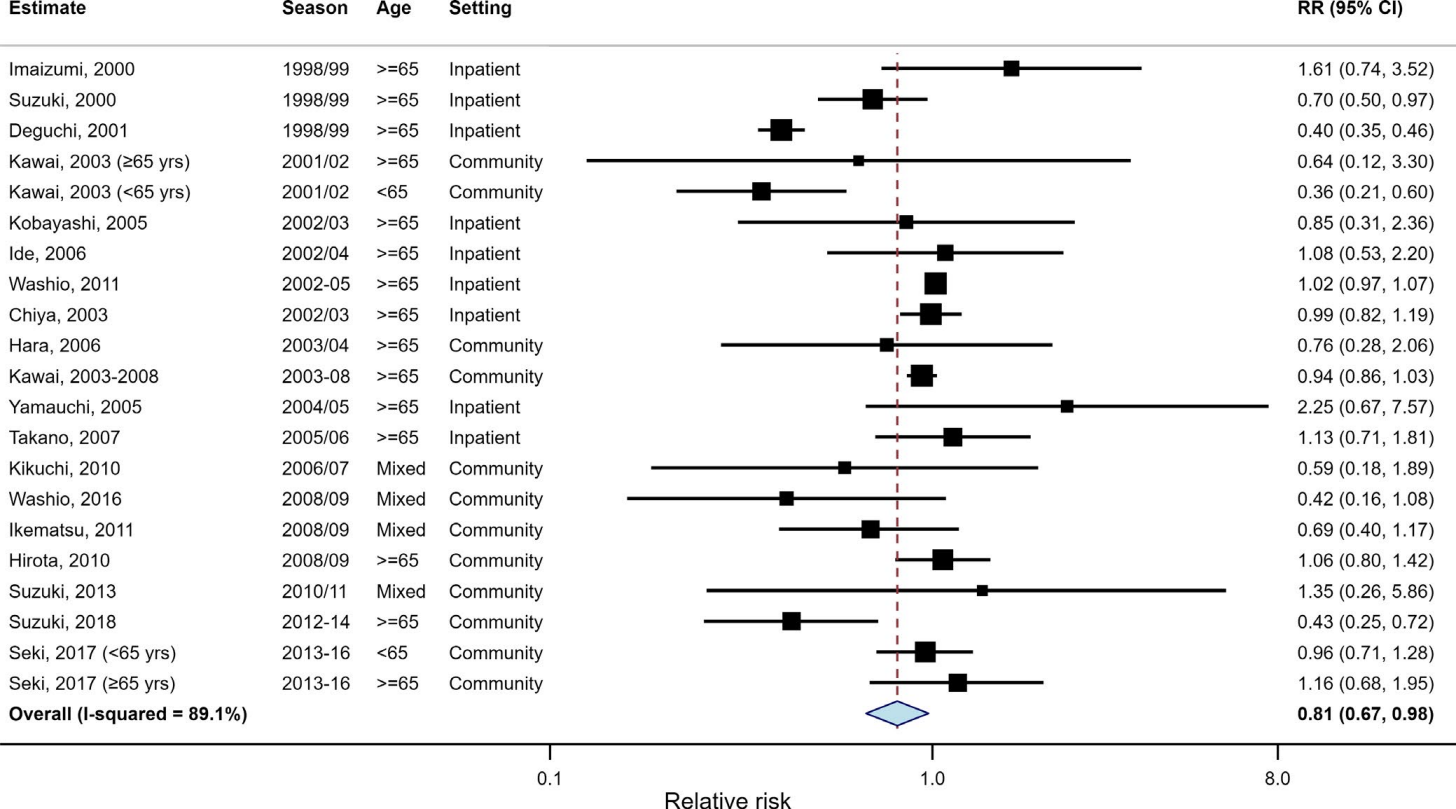
Forest plot of vaccine effectiveness estimates and the overall pooled estimate from a random‐effects model without adjustments (diamond). Boxes represent estimates weighted by the inverse of their variance, lines 95% CIs

After exclusions and stratification for age a total of 21 age‐specific RR estimates from seasons 1998/1999 ‐ 2015/2016 contained the necessary data for meta‐analysis (Figure 3). Most (15/21) were from studies of individuals aged ≥65; the majority were conducted in community settings (12/21) and used a cohort study design (16/21). In crude analysis, overall VE was 19.1% (95% CI: 2.3%‐33.0%) with significant heterogeneity between studies (I^2^: 89.1%; Figure 3). There was no evidence of significant publication bias from small studies (*P* = .327).

**Figure 4:**
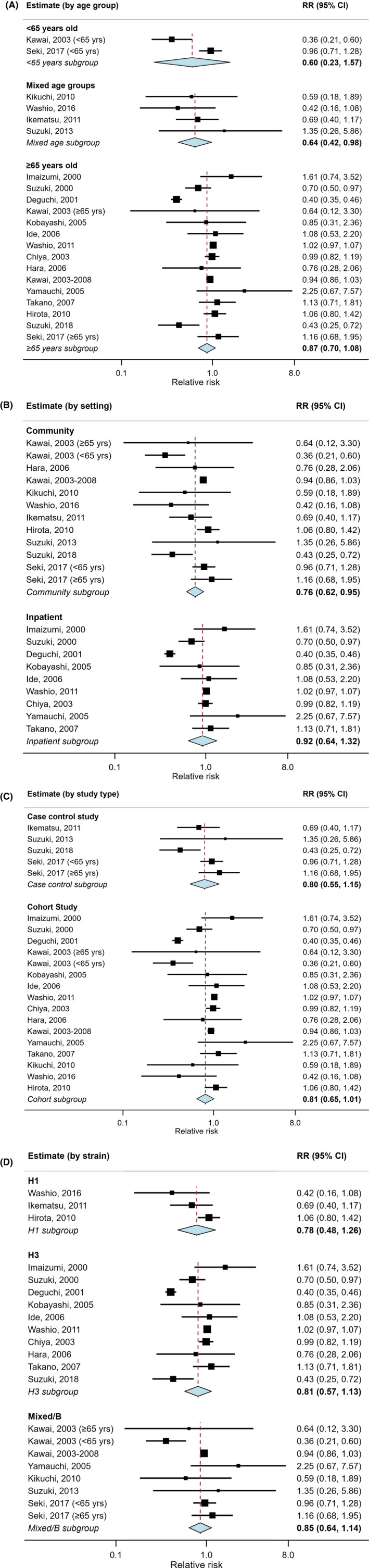
A‐D, Forest plot of vaccine effectiveness estimates stratified by (A) age group; (B) study setting; (C) study design and (D) circulating subtype. Diamonds represent pooled estimates per subgroup from a random‐effects model without adjustments. Boxes represent estimates weighted by the inverse of their variance, lines 95% CIs

Exploring heterogeneity, study design had no substantial impact on estimates with VE of 18.7% (−1.3 ‐ 34.7) in cohort and 20.3% (−15.5‐45.0) in case control studies (*P* < .93; Figure 4). A trend of decreasing VE with increasing age was observed with VE of 40.1% (−57.3‐77.2) in the <65 group; 35.7% (2.0‐57.8) in the mixed age group and 12.9% (−8.0‐29.8) in those aged ≥65; *P* = .21. Studies conducted at hospital or nursing home settings reported a lower VE of 7.8% (−32.4‐35.8) compared with 23.5% (5.0 ‐ 38.5) in studies conducted in the community (*P* = .37). Additionally, VE reported during seasons of H1 virus dominance was slightly higher (22.0%, 95% CI: −25.9‐51.7) than seasons of H3 (19.3%, −13.3‐42.6) or mixed/B virus subtype circulation (14.6%, −13.7‐35.9). None of these characteristics was significantly predictive of VE at the 95% confidence level.

### 3.3 Antiviral use in elderly patients

Fourteen observational studies reported on the effectiveness of antivirals (amantadine, oseltamivir, laninamivir, peramivir, and zanamivir) among elderly patients to prevent or treat various influenza‐related outcomes (Table 1). When used prophylactically in hemodialysis patients, oseltamivir prevented influenza (attack rate; 6.5% in untreated vs 0% in treated group, *P* < .01) ^34^ but post‐exposure administration of amantadine did not show significant reductions in fever duration following ILI in 1999.^75^ Finally, several studies assessed the average time for influenza cases to become afebrile following antiviral therapy. Oseltamivir was more effective when administered within 24 hours; and the proportion of afebrile patients reached to 30.8%, 80.8%, and 100% after 24, 48, and 72 hours of the initial administration, in the elderly aged 65 yrs and above.^76^ In all age groups including those over 65yrs, oseltamivir was more effective for type A than B in terms of average time to become afebrile; 40.4 ± 30.8 hours vs 51.8 ± 40.1 hours (*P* < .05).^72^ Combination oseltamivir and amantadine achieved similar reductions in the duration of fever in patients over 65yrs ^77^ and peramivir and oseltamivir also performed similarly with respect to the time to defervescence (30.9 ± 18.7 hours vs 34.7 ± 18.6 hours) or survival rate [95.7% (22/23) vs 100% (9/9), respectively].^78^

### 3.4 Health economic burden and cost‐effectiveness of preventive measures

We identified only one health economic study which directly assessed influenza costs. Sruamsiri et al, used a nationwide database of Japanese patients with a diagnosis of influenza admitted for at least 2 days between April 2014 and March 2015, and applied a structural equation modeling approach to estimate length of stay and total hospitalization costs.^78^

Influenza resulted in mean hospitalizations of 11.3 days for patients aged 16‐64 years, rising to 16.1 days in patients aged ≥65 years and mean costs of 609,196 JPY in younger adults; and 715,614 JPY in elderly patients (≥65 years). Intensive care hospitalization costs were approximately double. Underlining the significant resource consumption in elderly and comorbid patient groups, a retrospective peramivir study between 2012 to 2015 followed elderly inpatients (≥65 years) with influenza and existing comorbidities. Mean length of hospitalization was 14.0 days, with a range from 3‐155 days.^52^

To assess post‐infection socio‐economic outcomes, an observational study in outpatients aged >50 years confirmed the etiology of viral infection and compared them in patients with influenza, and those without.^46^ Days of absence from work (3.1 vs 2.2 days), days of reduced activity (5.2 vs 3.6), caregiver absences, and impacts on daily activity were significantly higher in influenza patients, documenting the broader societal impact of influenza beyond direct medical costs.

Three simulation studies on the cost‐effectiveness of influenza vaccination or seasonal influenza prophylaxis were identified.^16^, ^47^, ^79^ Influenza vaccination resulted in a cost‐effectiveness ratio of 516,332 Japanese yen per year of life saved (JPY/YOLS).^47^ Simultaneous influenza and pneumococcal vaccination provided a favorable ratio (459,874 JPY/YOLS) suggesting synchronized prophylaxis was more cost‐effective. The cost‐effectiveness of TIV to QIV switch in Japan was also studied.^16^ QIV‐use yielded 0.73 YOLS per 100 000 inhabitants annually (95% CI, 0.72‐0.75), reduced spending by 9,435,360 JPY per 100,000 inhabitants and was consequently found to be cost‐effective from both payer and societal perspectives.

A pharmacoeconomic evaluation compared influenza prophylaxis using an oral neuraminidase inhibitor (either for 7 days as post‐contact prophylaxis or via seasonal administration for 6 weeks), or vaccine.^79^ Seven day post‐contact prophylaxis resulted in better health outcomes and lower costs in comparison with no prophylaxis. Vaccination resulted in lower costs and superior health outcomes compared with seasonal prophylaxis; the authors therefore suggested seasonal prophylaxis treatment may be a supplemental strategy to prevent influenza infections.

## 4 DISCUSSION

This systematic review and meta‐analysis summarized the existing evidence from Japan on influenza‐related epidemiology and economics and described the reported effectiveness of influenza vaccines. The underlying body of existing literature is substantial, with >110 papers describing studies conducted across seasons, geography and disciplines. The primary feature of these studies was their heterogeneity: the reported influenza incidence rate, for example, varied from a low of 0.7% to a high of over 55% per year. Contributors to this variation may include differences in study population, setting, and influenza activity as exemplified by a 1998/99 study, conducted in a season of changing influenza strains, vaccine mismatch, and outbreaks in institutional settings where high‐risk populations with comorbidities or complications are grouped, leading to high attack rates.^80^ However, the magnitude of this range seems unexplainable by normal epidemiological variation and it is more likely that case definitions of differing sensitivity and specificity, used in individual studies, are responsible. Laboratory‐confirmed influenza cases make up only a fraction of all ILI episodes ^9^ and it is therefore inappropriate to make direct comparisons between incidence rates without considering the context and methods of the underlying studies. These data confirm that very high attack rates of symptomatic illness are possible within residential care facilities during outbreaks but are not representative of broader influenza infection rates within the community.

Sentinel disease surveillance is designed to provide a more consistent picture of epidemiological variation, but Japanese authors have proposed that included medical institutions may not be fully representative of national incidence, due to stringent eligibility criteria. A more comprehensive system has been developed to improved accuracy of epidemiological research, which includes auxiliary outpatient health data and the incorporation of age‐stratified denominators for incidence calculations, may benefit the accuracy of future epidemiological research.^81^ This method provides updated national influenza estimates of 9 910 000, 10 460 000, 14 580 000 in the 2015/16, 2016/17, 2017/18 seasons.

A focus of this review was to understand consequences of influenza in the elderly and although the incidence of reported influenza is highest in children, the disease burden in terms of hospitalizations and deaths appears to be highest in individuals aged >60 years.^6^, ^23^, ^30^, ^45^ This is because some older individuals have weakened immune systems, are more susceptible to infection, and are likely to suffer from comorbid conditions which place them at higher risk of requiring hospitalization, more extensive clinical support and suffering serious medical outcomes including death.^12^ This is consistent with influenza excess mortality studies from other East‐Asian countries which find that >85% of influenza deaths are in individuals aged ≥65 years.^82^, ^83^, ^84^

Japanese data describing influenza in patients with underlying medical conditions were sparse, though elderly groups remain priority target groups for vaccination.^85^ Globally, there is increasing evidence supporting an association between influenza infection and non‐pulmonary disease including cardiac, neurologic, endocrine, and other complications ^86^ which are more common in people with underlying risk factors. This may be an avenue for future research in Japanese populations. In contrast, the severe respiratory complications of influenza infection, such as pneumonia, are recognized in Japan and their treatment has correspondingly been incorporated into influenza management guidelines, especially in care facilities.

WHO recommends vaccination as the most effective prevention method for influenza in people ≥65 years ^14^ and Japan achieves one of the highest vaccine coverage rates in Asia, with ~50% of the elderly population vaccinated annually.^87^ Over 20 studies were identified which described VE or provided the relevant data to calculate it, allowing for a meta‐analysis. While the analysis included heterogeneous studies, it demonstrated a protective trend in vaccinated groups, reinforcing the value of influenza vaccination. However, the overall VE reported in these studies was rather low at 19% (2% ‐ 33%), and slightly lower than reported in global influenza vaccine effectiveness meta‐analyses.^17^, ^88^ This may because of the high proportion of studies conducted in the elderly population in whom VE is likely to be low.

We identified a non‐significant trend of lower VE in older individuals; VE in those younger than 65 was 40% compared to 13% in those ≥65. These results are similar to those observed in a meta‐analysis of case control and cohort studies conducted in a Western elderly population identifying VE of 25% in reducing visits (95% CI: 6‐40%) and a 14% reduction in hospitalizations (95% CI: 7‐21%; *P* < .001).^18^ They are also broadly consistent with international studies showing marginal VE among older adults ^89^ although confounding by circulating viral subtypes may also be contributing.^90^, ^91^ Studies conducted at inpatient facilities also reported lower VE, but all nine studies in inpatient settings included only individuals aged ≥65. Despite these limitations, the lower VE observed in older adults and from inpatient settings is concerning because in Japan, as elsewhere, the elderly and vulnerable populations suffer a disproportionate burden of influenza hospitalization and mortality.

Few articles described the costs of influenza treatment and only weak evidence suggested influenza costs were higher among the unvaccinated elderly. Most economic studies examined treatments employed over different seasonal influenza epidemics and therefore provided a limited picture of the economic burden; additional studies, particularly using representative healthcare databases, would be welcome. Limited economic assessments of influenza interventions indicated vaccination is a cost‐effective preventative health practice in the elderly using currently available vaccines.

## 5 LIMITATIONS

Some relevant grey literature may have been overlooked during the search for reasons of practical feasibility. A study quality assessment was not conducted, therefore these results should be viewed as representative of the existing literature on this topic. The timeframe of the review excluded publications addressing effectiveness of baloxavir, a new antiviral drug, and no distinction was made between time periods when trivalent and quadrivalent influenza vaccines (before/after 2015/2016 season) were in use. A meta‐analysis found considerable heterogeneity between VE studies that examined VE, and we employed only a simple meta‐regression approach which failed to identify strong deterministic variables. Administration of antiviral prophylaxis in institution‐based VE studies was also not captured, leading to incorporation of potentially biased VE estimates. The meta‐analysis was also subject to methodological limitations: we used crude RRs where adjusted, age‐stratified effect sizes were unavailable and made assumptions to convert ORs to RRs. Most included studies were observational cohorts, a design vulnerable to confounding based on the baseline health status of individuals and systematic biases affecting our results cannot be excluded.^92^ Here, a large difference in VE according to circulating type or subtype was not observed, but we used national‐level surveillance data which may not have been reflective of local epidemiology at the sites where studies were conducted.

## 6 CONCLUSION

This review of English and Japanese literature represents the first comprehensive synthesis of the seasonal influenza literature in Japan. The highest burden of hospitalizations and deaths was in the elderly population. Published data suggest influenza vaccines are effective, but with suboptimal VE in the elderly and institutionalized individuals most at risk. Additional research, particularly into the health economics of different influenza management tools, is needed to maximize positive health outcomes in the growing, elderly Japanese population.

## CONFLICT OF INTEREST

Joshua Nealon, Yuriko Hagiwara are employed by Sanofi Pasteur, a company which produces influenza vaccines. Daisuke Tsuzuki and Marwa Klai were working at Sanofi at the time of performing the study.

## AUTHOR CONTRIBUTIONS

**Kiyosu Taniguchi:** Supervision (lead); Writing‐review & editing (equal). **Shunya Ikeda:** Supervision (lead); Writing‐review & editing (equal). **Yuriko Hagiwara:** Conceptualization (equal); Data curation (equal); Formal analysis (equal); Investigation (equal); Methodology (equal); Project administration (lead); Resources (equal); Supervision (equal); Writing‐original draft (equal); Writing‐review & editing (lead). **Daisuke Tsuzuki:** Conceptualization (supporting); Writing‐review & editing (equal). **Marwa Klai:** Formal analysis (equal); Investigation (equal); Software (equal). **Yoko Sakai:** Data curation (equal); Formal analysis (equal); Investigation (equal); Methodology (equal); Project administration (lead); Resources (equal); Visualization (equal); Writing‐original draft (supporting). **Bruce Crawford:** Formal analysis (equal); Investigation (equal); Methodology (equal); Validation (equal); Writing‐original draft (supporting). **Joshua Nealon:** Conceptualization (lead); Formal analysis (equal); Funding acquisition (lead); Methodology (equal); Supervision (lead); Writing‐original draft (equal); Writing‐review & editing (lead).

## Supporting information

Table S1‐S3
