# Supplementary material for: Epidemiology and burden of illness of seasonal influenza among the elderly in Japan: A systematic literature review and vaccine effectiveness meta‐analysis
Source: Influenza Other Respir Viruses. 2020 Sep 30;15(2):293–314. doi: 10.1111/irv.12814 (PMC7902263; doi:10.1111/irv.12814)
Supplement: Supplementary file 1 — Table S1‐S3 [file IRV-15-293-s001.docx]

# Supplementary Table 1

**Seasonal influenza search strategy (ICHUSHI)**

| **Search Number** | **Search String** |
| --- | --- |
| ***Indication*** | |
| 1. | インフルエンザ-ヒト/TH OR インフルエンザ/TA OR 季節性インフルエンザ/TA OR インフルエンザ感染/TA OR インフルエンザウィルス/TA OR インフルエンザ様疾患/TA |
| ***Outcomes*** | |
| 2. | 疫学/TH OR 臨床背景/TH OR 共存疾患/TH OR 併存疾患/AL OR 発生率/TH OR 有病率/TH OR 死亡率/TH OR 患者重症度/TH OR 重症度/AL OR 費用と費用分析/TH OR 費用/AL OR 医療費/TH OR 費用効果分析/TH OR 医療経済/TH OR 医療経済/AL OR 質調整生存年/TH OR 障害調整生存年/TH OR 費用対効果/TA OR 財政/TA OR 効用/TA OR 負担/TA OR 経済的負担/TA OR 経済評価/TA OR QALY/TA OR DALY/TA OR 費用対効用/TA OR 発症率/TA OR 超過死亡/TA OR 接種率/TA OR 間接効果/TA OR 薬剤耐性/TA OR 予防効果/TA OR 予防投与/TA OR 抗ウィルス/TA OR 抗ウィルス剤/TH |
| ***Demographic*** |  |
| 3. | ((CK=ヒト) AND (CK=高齢者(65～),高齢者(80～))) OR 成人/TA OR 中年/TA OR 高齢/TA |
| ***Japanese adult patients with seasonal influenza and relevant outcomes*** | |
| 4. | #1 AND #2 AND #3 |
| ***Combined with limits*** | |
| 5. | #4 AND (LA=日本語,英語) AND (DT=1997/1/1:2018/11/20) AND (AB=Y) AND (PT=原著論文) |

TH: Indexed terms based on the medical thesaurus; Similar to the MeSH function in the PubMed database

TA: Identifies all applicable search terms including partial matches, within the title and abstract

SH: Identifies search terms with the designated subcategory index tag

CK: Check tag, which limits searches from a specific perspective

LA: Publication language restrictions

DT: Publication year restrictions

AB: Abstract availability (Yes/No)

PT: Restricts publication type to designated categories

# Supplementary Table 2

**Seasonal influenza search strategy (PubMed)**

| **Search Number** | **Search String** |
| --- | --- |
| ***Indication*** | |
| 1. | influenza[MeSH Terms] or "seasonal influenza"[tiab] or influenza[tiab] or “influenza like illness”[tiab] |
| ***Outcome*** | |
| 2. | epidemi*[tiab] or “analyses, demographic”[MeSH Terms] or demographic*[tiab] or inciden*[tiab] or prevalen*[tiab] or mortality*[tiab] or strain*[tiab] or burden[tiab] or severity[tiab] or cost-effectiveness[tiab] or cost[tiab] or “cost, treatment”[MeSH Terms] “health care resource”[tiab] or “burden of illness”[MeSH Terms] or “burden of illness”[tiab] or “analyses, cost”[MeSH Terms] or DALY[tiab] or QALY[tiab] or utility[tiab] or cost-utility[tiab] or effectiveness[tiab] or “excess mortality”[tiab] or “coverage rate”[tiab] or “vaccination rate”[tiab] or “herd immunity”[tiab] or “antiviral resistance”[tiab] or AMR[tiab] or “preventive effect”[tiab] or “indirect vaccine effect”[tiab] or (antivirals[tiab]) or (((antiviral agents[MeSH Terms]) or antivirals[MeSH Terms]) |
| ***Demographic*** |  |
| 3. | (elderly OR adult) AND Japan* |
| ***Japanese adult patients with seasonal influenza and relevant outcomes*** | |
| 4. | #1 AND #2 AND #3 |
| ***Combined with limits*** | |
| 5. | #4 AND “1997/01/01"[PDAT] : "2018/11/20"[PDAT] and (English[lang] OR Japanese[lang]) |

MeSH: Stands for Medical Subject Headings, which is the National Library of Medicine's controlled vocabulary thesaurus used for indexing journal articles

TIAB: Identifies specified search terms within the article’s title and abstract

*: Truncation unlimited

PDAT: Filters articles with only the published timeframe of interest

Lang: Filters articles with only the languages of interest

# Supplementary Table 3

**Seasonal influenza search strategy (EMBASE)**

| **Search Number** | **Search String** |
| --- | --- |
| ***Indication*** | |
| 1. | 'seasonal influenza':ab,ti or 'influenza'/exp or 'influenza':ab,ti or ‘influenza virus’:ab,ti or ‘influenza like illness’:ab,ti |
| ***Outcomes*** | |
| 2. | 'epidemiological data':ab,ti OR 'demography'/exp OR ‘demography’:ab,ti OR ‘bacterial strain’/exp OR ‘disease severity’/exp OR 'prevalence':ab,ti OR 'incidence':ab,ti OR 'mortality':ab,ti OR ‘clinical effectiveness’/exp OR ‘excess mortality’:ab,ti OR ‘coverage rate’:ab,ti OR ‘vaccination rate’:ab,ti or ‘herd immunity’:ab,ti or ‘antiviral resistance’:ab,ti or AMR:ab,ti or ‘preventive effect’:ab,ti or `indirect vaccine effect’:ab,ti or antiviral:ab,ti or ‘antiviral agent’:ab,ti |
| 3. | ((qaly OR quality) AND adjusted) OR ((daly OR 'disability'/exp) AND adjusted) OR 'cea'/exp OR cba OR cua OR (economic NEAR/10 model*) OR 'markov model' OR 'decision tree'/exp OR 'decision analytic model' OR 'discrete event model' OR 'discrete event simulation' OR 'economic evaluation'/exp OR 'economic evaluation' OR 'cost benefit'/exp OR 'cost benefit' OR 'cost effectiveness'/exp OR 'cost effectiveness' OR 'cost minimization'/exp OR 'cost minimization' OR 'cost utility'/exp OR 'cost utility' OR 'budget impact' OR ‘cost’/exp OR cost* OR ‘health care cost’ OR ‘health economics’/exp OR ‘health economics’ OR ((cost/exp OR cost* OR burden) AND (illness/exp OR illness OR disease/exp OR disease OR sickness/exp OR sickness)) OR (cost* AND (indirect OR ‘nonmedical’ OR medical OR direct OR health OR inpatient OR hospital* OR outpatient OR physician OR drug OR ‘adverse event’ OR management)) |
| ***Population*** | |
| 4. | ([adult]/lim OR [middle aged]/lim OR [aged]/lim OR [very elderly]/lim) AND [humans]/lim AND ('japanese (people)' OR japan*) |
| ***Japanese adult patients with seasonal influenza and relevant outcomes*** | |
| 5. | #1 AND (#2 OR #3) AND #4 |
| ***Combined with limits*** | |
| 6. | ([article]/lim OR [article in press]/lim) AND ([english]/lim OR [japanese]/lim) AND [abstracts]/lim AND [1997-2018]/py AND [embase]/lim |

exp: Maps search terms to the Emtree preferred indexing term and then searches for the related narrower or child terms

ab, ti: Finds designated syntax in the title and abstract

py: Filters articles by publication year range

la: Filters articles by the published language

lim: Restricts searches based on publication type, language and availability of abstract, and database source

*: Truncation unlimited for the designated syntax
